# DAXX co-folds with H3.3/H4 using high local stability conferred by the H3.3 variant recognition residues

**DOI:** 10.1093/nar/gku090

**Published:** 2014-01-31

**Authors:** Jamie E. DeNizio, Simon J. Elsässer, Ben E. Black

**Affiliations:** ^1^Department of Biochemistry and Biophysics, Graduate Program in Biochemistry and Molecular Biophysics, Perelman School of Medicine, University of Pennsylvania, Philadelphia, PA 19104-6059, USA and ^2^MRC Laboratory of Molecular Biology, Cambridge CB2 0QH, UK

## Abstract

Histone chaperones are a diverse class of proteins that facilitate chromatin assembly. Their ability to stabilize highly abundant histone proteins in the cellular environment prevents non-specific interactions and promotes nucleosome formation, but the various mechanisms for doing so are not well understood. We now focus on the dynamic features of the DAXX histone chaperone that have been elusive from previous structural studies. Using hydrogen/deuterium exchange coupled to mass spectrometry (H/DX-MS), we elucidate the concerted binding-folding of DAXX with histone variants H3.3/H4 and H3.2/H4 and find that high local stability at the variant-specific recognition residues rationalizes its known selectivity for H3.3. We show that the DAXX histone binding domain is largely disordered in solution and that formation of the H3.3/H4/DAXX complex induces folding and dramatic global stabilization of both histone and chaperone. Thus, DAXX uses a novel strategy as a molecular chaperone that paradoxically couples its own folding to substrate recognition and binding. Further, we propose a model for the chromatin assembly reaction it mediates, including a stepwise folding pathway that helps explain the fidelity of DAXX in associating with the H3.3 variant, despite an extensive and nearly identical binding surface on its counterparts, H3.1 and H3.2.

## INTRODUCTION

Nucleosome assembly is a dynamic multi-step process that is regulated by histone chaperones. Tailored to their highly conserved architectural and regulatory function within the nucleosome, histone protomers outside of the nucleosomal context require a diverse family of proteins to prevent unspecific contacts. These so-called histone chaperones use a variety of structural motifs to contact their cognate histones and seem to have largely non-overlapping specific functions in histone metabolism and nucleosome assembly in distinct chromosomal loci (1). The trend for functional specialization in higher eukaryotes is particularly apparent among a subclass of histone chaperones, including DAXX, HIRA and HJURP that selectively bind histone H3 variants H3.3 (DAXX and HIRA) and CENP-A (HJURP) (2–9). All of these variant pathways are distinct from the CAF1 pathway for canonical histones H3.1 and H3.2 (3,10). The unique structures and binding mechanisms of these histone chaperone complexes are expected to reflect their specific functions and are the subject of current investigation.

DAXX is a metazoan histone chaperone, specific to the evolutionarily conserved histone variant H3.3. Its function is tied to telomeric and centromeric heterochromatin, where replication-independent deposition of H3.3 appears to serve a role in chromatin maintenance, epigenetic and genetic stability and tumor suppression (8,9,11–13). Thus, understanding the molecular function of DAXX will help pinpoint its role in health and disease. DAXX protein encompasses an N-terminal 4-helix bundle (14), a central histone binding domain (HBD) (8) and a C-terminal domain that is predicted to be disordered. Co-crystal structures of the H3.3/H4/DAXX HBD complex revealed an extended fold of the DAXX HBD that envelops an H3.3/H4 dimer with seven consecutive α helices (15,16). Interaction of the overall basic DAXX HBD domain with the Lys/Arg-rich histones is dominated by localized salt bridges and large hydrophobic interfaces. Given the extensive interaction surface, it is remarkable that DAXX selectively chaperones histone variant H3.3 *in vivo* by reading out minor amino acid differences from canonical histones H3.1/2. The varying residues are primarily confined to a small region, from amino acids 87 to 90 of H3.3, and are buried in the histone chaperone complex. Experiments with purified components shed some light into the mode of discrimination revealing that the N-terminal helices (α1 and α2 helices) of the DAXX HBD, also termed ‘tower’, disfavor side chain substitutions at H3.3 Gly90 (such as Met90 in H3.2) through water-mediated contacts. However, considering the binding energy gained over the vast histone chaperone interface in comparison with that contributed by H3.3-specific residues—and the absence of clear ‘lock-and-key’ recognition—it has remained elusive how high specificity could be achieved.

Recent studies using H/DX-MS revealed conformational flexibility in the histone fold (17–20) that could be exploited in the case of variant-specific recognition by chaperones. H/DX-MS measures the exchange of amide protons along the polypeptide backbone with deuterons from heavy water. H/DX is fast in unfolded regions of proteins and slow in regions with stable folded structure, where the amide protons are engaged in hydrogen bonds (21). Thus, it is an ideal technique to monitor the conformational changes of histone chaperone complexes.

Here we use H/DX-MS to determine the nature of the H3.3/H4/DAXX heterotrimer in solution. We measure the stability conferred to both the histone substrate complex ([H3.3/H4]_2_) and the monomeric chaperone on heterotrimer formation, test the degree to which DAXX-binding can induce a stable fold to a partially unfolded H3.3 mutant protein and compare the backbone dynamics of DAXX when bound to its target variant (H3.3) versus when bound to an inappropriate substrate (H3.2). Considering these findings, we propose a model for how selectivity is achieved in the DAXX-mediated nucleosome assembly pathway for the H3.3 variant.

## MATERIALS AND METHODS

### Protein expression and purification

Vectors, expression and purification conditions for histones and DAXX HBD (residues 183–417; hereafter referred to as DAXX) were essentially as previously described (15). Briefly, bacterial expression of histones and DAXX was individually expressed in BL21 Star(DE3) cells (Invitrogen) into inclusion bodies for 4–6 h at 37°C. Inclusion bodies were resolubilized in 6 M guanidine-HCl, 1 M NaCl, 50 mM Tris–HCl, pH 8, and purified on a Ni-NTA affinity column. To prepare (H3/H4)_2_ heterotetramers or DAXX-histone complexes, equimolar ratios of all proteins were mixed in 6 M guanidine-HCl, 50 mM MOPS, pH 7, 0.5 M NaCl, 5 mM EDTA, 10% glycerol and dialyzed against 50 mM MOPS, pH 7, 0.5 M NaCl, 1 mM EDTA, 10% glycerol over 24–48 h, exchanging dialysis buffer at least once. Refolding reactions were spun for 30 min at 30 000rpm to remove insoluble material, and supernatants were loaded onto a Superdex 200 column in 10 mM MOPS, pH 7, 0.5 M NaCl, 1 mM DTT, 0.2 mM PMSF to yield the final complex. Note that at low-salt concentrations or at low concentrations of histones without DAXX, H3/H4 heavily populates a dimer state (22), whereas under the conditions we used—designed to allow a side-by-side comparison to the H3/H4/DAXX trimer complexes—H3/H4 more heavily populates a tetramer state. To prepare the DAXX monomer, lyophilized protein was resuspended in 6 M guanidine-HCl, 50 mM MOPS, pH 7, 0.5 M NaCl, 1 mM EDTA, 5 mM DTT, 10% glycerol, dialyzed against two changes of 50 mM MOPS, pH 7, 0.5 M NaCl, 1 mM DTT, 0.2 mM PMSF and finally dialyzed into 10 mM MOPS, pH 7, 0.5 M NaCl, 1 mM DTT, 0.2 mM PMSF. The refolding reaction was spun for 10 min at 15 000rpm, and soluble DAXX (yield ∼11 µM) was used directly in the H/DX reaction.

### H/DX reactions

Deuterium on-exchange was carried out on ice by adding 5 µl of protein sample (2–10 µg of protein or protein complex) to 15 µl of deuterium on-exchange buffer (5 mM H_2_NaO_4_P/HNa_2_O_4_P pD 7, 0.5 M NaCl in D_2_O) so that the final D_2_O content was 75%. At each indicated time point, the exchange mixture was added to 30 µl quench buffer (1.66 M guanidine-HCl, 0.8% formic acid, 10% glycerol in H_2_O) on ice and immediately frozen in liquid nitrogen. The samples were stored at −80°C until analysis by MS.

### Protein fragmentation and MS

H/DX samples were individually thawed at 0°C for 2 min, then injected (50 µl) and pumped through an immobilized pepsin (Sigma) column at an initial flow rate of 50 µl/min for 2 min followed by 150 µl/min for 2 min. Pepsin (Sigma) was immobilized by coupling to POROS 20 AL support (Applied Biosystems) and packed into column housings of 2 mm × 2 cm (64 µl) (Upchurch). Protease-generated fragments were collected onto a C18 HPLC trap column (800 µm × 2 mm, Dionex). Peptides were eluted into and through an analytical C18 HPLC column (0.3 × 75 mm, Agilent) by a linear 12–55% buffer B gradient over 15 min at 6 µl/min (Buffer A: 0.1% formic acid; Buffer B: 0.1% formic acid, 99.9% acetonitrile). The effluent was electrosprayed into the mass spectrometer (LTQ Orbitrap XL, Thermo Fisher Scientific). The SEQUEST (Bioworks v3.3.1) software program (Thermo Fisher Scientific) was used to identify the likely sequence of the parent peptides using non-deuterated samples via tandem MS.

### H/DX data analysis

MATLAB-based MS data analysis tool—ExMS—was used for data processing (23). Briefly, the ExMS program searches raw MS data, identifies individual isotopic peaks/envelopes from a list of MS/MS peptides obtained from SEQUEST search and calculates centroid values of these envelopes. The program is used to first identify the isotopic envelope centroid and chromatographic elution time of each parental non-deuterated peptide, and then this information is subsequently used to identify deuterated peptides.

The level of H/DX at each time point is expressed as either the percent exchange or number of deuterons within each peptide. First, each individual deuterated peptide is corrected for loss of deuterium label during H/DX-MS data collection (i.e. back exchange after quench) by normalizing to peptides from a ‘fully-deuterated’ reference sample. These reference samples are prepared in 75% deuterium to mimic the on-exchange experiment, but under acidic denaturing conditions (0.5% formic acid), and incubated overnight so that each amide proton undergoes full exchange. The centroid values for the experimental datasets prepared under native conditions are then normalized to the maximal deuterium incorporation we can measure for each peptide to calculate exchange levels. Using the ‘fully-deuterated’ samples and accounting for all the peptides in our data sets, our typical back-exchange average per peptide for an entire data set was 18%, which is within close range of some of the lowest deuterium losses reported for other proteins in H/DX-MS (24). Calculation of deuterium loss correction and other data operations were performed using MATLAB. In addition, maps of rate-classes along the polypeptide were assembled using the H/DX data as described (18,25). In some instances, helical segments known from the H3.3/H4/DAXX heterotrimer (PDB 4H9N) were used to first place the slowest exchanging positions.

### Size exclusion chromatography–multi-angle light scattering

Multi-angle light scattering (MALS) was measured in line with an HPLC size exclusion chromatography (SEC) setup. In particular, a DAWN HELEOS DLS (Wyatt) instrument was directly connected to an Agilent 1100 HPLC system equipped with a Superdex 200 GL 10/300 column (Amersham). 500 µg of each sample (5 mg/ml) was injected, and DLS signals were recorded for 25 ml at a flow rate of 0.5 ml/min.

## RESULTS

### All histone fold helices of H3.3 and H4 are stabilized upon DAXX binding

**Figure 1.**
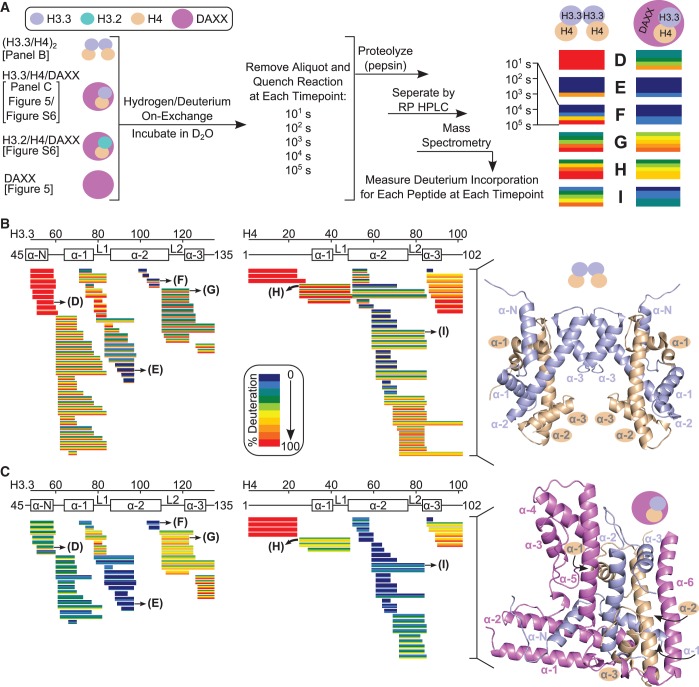
H3.3/H4 dimer is globally stabilized by DAXX upon heterotrimer formation. (**A**) Experimental scheme for comparing H/DX of (H3.3/H4)_2_ heterotetramer, H3.3/H4/DAXX heterotrimer complex, H3.2/H4/DAXX heterotrimer complex and DAXX monomer. The locations of the ribbon diagrams with all time points for each corresponding H/DX data set are listed. (**B** and **C**) H/DX data for the histones from (H3.3/H4)_2_ and H3.3/H4/DAXX. Each horizontal bar represents an individual peptide from (H3.3/H4)_2_ (B) or H3.3/H4/DAXX (C) and is color-coded for percent deuteration at each time point (10^1^, 10^2^, 10^3^, 10^4^ and 10^5^ s) by individual stripes within each bar. Peptides are placed beneath schematics of the secondary structural elements of H3.3 or H4 from the crystal structures of (H3.3/H4)_2_ (B) [from the H3.3 nucleosome, PDB 3AV2; (27)] and H3.3/H4/DAXX (C) [PDB 4H9N; (15)], which are shown adjacent to the H/DX data from each respective complex. (**D–I**) Enlarged peptides from panel B (D–I, left) and panel C (D–I, right) are shown side-by-side.

To measure the backbone dynamics of free histones and histone chaperone complexes, we used H/DX-MS. The (H3.3/H4)_2_ heterotetramer and H3.3/H4/DAXX heterotrimer were incubated in D_2_O (heavy water) separately on ice to exchange the amide protons on the peptide backbone with deuterons (Figure 1A). The low exchange temperature was chosen to extend backbone exchange over a longer time course because a previous study showed that at room temperature (H3/H4)_2_ heterotetramers are completely exchanged at all locations by 10^3^ s (18). Even with the ∼10-fold slower chemical exchange rates (21) and greater thermal stability gained by performing experiments on ice, the (H3.3/H4)_2_ heterotetramer is nearly entirely exchanged by 10^5^ s (Figure 1B). The samples were ‘quenched’ at each time point, ranging from 10^1^–10^5^ s, lowering the pH to the point (pH ∼2.3) at which the chemical exchange rate, ‘back-exchange’ in this case, is slowed so that it is negligible before MS measurements. The proteins were digested by pepsin, and deuterium incorporation on each resulting peptide fragment was measured by mass spectrometry (Figure 1A). H/DX of overlapping peptides was successfully measured at each time point for 97–99% of the histone fold domain (Figure 1B and C).

Fifty-eight peptides are identical in sequence between the (H3.3/H4)_2_ heterotetramer (Figure 1B) and H3.3/H4/DAXX heterotrimer (Figure 1C) samples, making possible ideal side-by-side comparisons (Figure 1D–I). For many of these peptides, mass spectra were obtained for multiple charge states, which further strengthen the confidence in our measurements. In 23 of the 58 unique peptides, it takes at least 1000 times as long to achieve the same level of H/DX in the H3.3/H4/DAXX heterotrimer as compared with in the (H3.3/H4)_2_ heterotetramer (Supplementary Figure S1B); in 26 of the 58 unique peptides it takes 10–1000 times as long (Supplementary Figure S1C). In total, only 9% of the histone peptides from the H3.3/H4/DAXX heterotrimer achieve >90% H/DX by 10^5^ s (Figure 1C). Besides the N-terminal tail of H4, which completely exchanges in both complexes, the sole exceptions to increased protection upon DAXX binding are seen in peptides spanning H3.3 L2 between the α2 and α3 helices (within residues 109–126), where H/DX is increased in the H3.3/H4/DAXX heterotrimer relative to the (H3.3/H4)_2_ heterotetramer at early time points (Figure 1B, C and G and Supplementary Figure S2). Overall, these H/DX data show that the extensive contacts established by DAXX around H3.3/H4 transmit global stability that prevents the transient unfolding of each histone.

### H3.3 αN helix adopts a stable fold when in complex with DAXX

**Figure 2.**
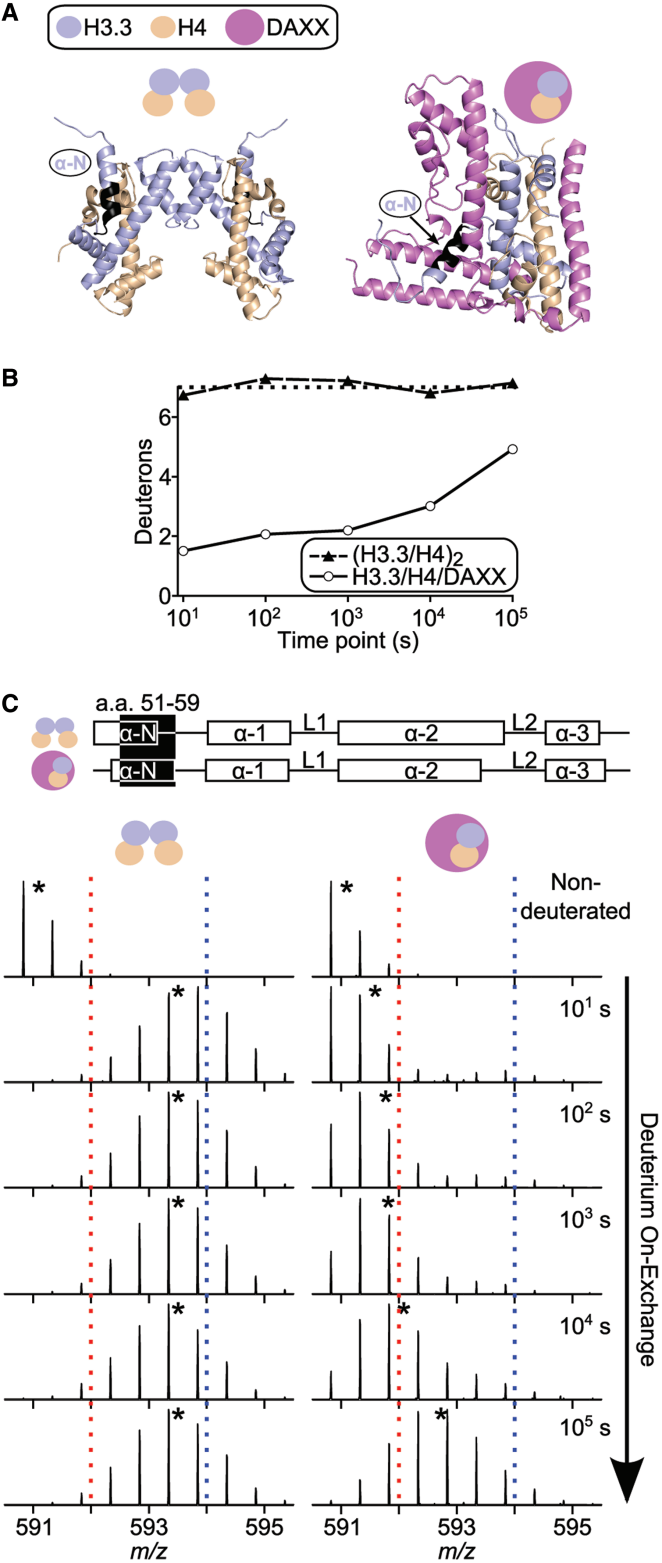
H3.3 αN helix is stably folded in the H3.3/H4/DAXX heterotrimer complex. (**A**) The location of a H3.3 peptide (residues 51–59), spanning the αN helix, is shown in black on both the (H3.3/H4)_2_ (PDB 3AV2) and H3.3/H4/DAXX (PDB 4H9N) crystal structures. The heterotetramer structure is from the stable secondary structures existing within the nucleosome core particle (27,28), but our data indicate the αN helix of H3.3 is unfolded in heterotetramers in solution. (**B**) Comparison of H/DX for the peptide spanning residues 51–59 from both complexes over the time course. The maximum number of deuterons possible to measure by H/DX is shown by a black dotted line. (**C**) Side-by-side analysis of MS data for the indicated peptide from (H3.3/H4)_2_ (left) or H3.3/H4/DAXX (right). Dotted red and blue lines serve as guideposts to highlight the differences in *m/z* shifts between the two complexes. Black stars denote the centroid locations.

The greatest local increase in protection from H/DX of the histones upon DAXX binding occurs in the region of the αN helix of H3.3 (Figures 1B–D and 2 and Supplementary Figure S2). In nucleosomes, the (H3/H4)_2_ heterotetramer exists with a stable αN helix at the DNA entry/exit site (26,27). Before nucleosome incorporation, however, this helix has rapid H/DX, indicating that in (H3/H4)_2_ tetramers in solution, it does not exist as a stable helix (18). In agreement with this, in (H3.3/H4)_2_ heterotetramers, the peptides spanning a.a. residues 48-61 achieve complete H/DX by the first time point (10^1^ s; a representative peptide is shown in Figure 2). Upon binding DAXX, the αN helix of H3.3 requires >10 000 longer timescales to achieve the same level of deuterium incorporation as compared with the (H3.3/H4)_2_ heterotetramer (Figures 1D and 2). Thus, the H3.3 αN helix adopts a stable fold upon binding DAXX.

### DAXX induces alterations to the α2-L2-α3 region of H3.3

**Figure 3.**
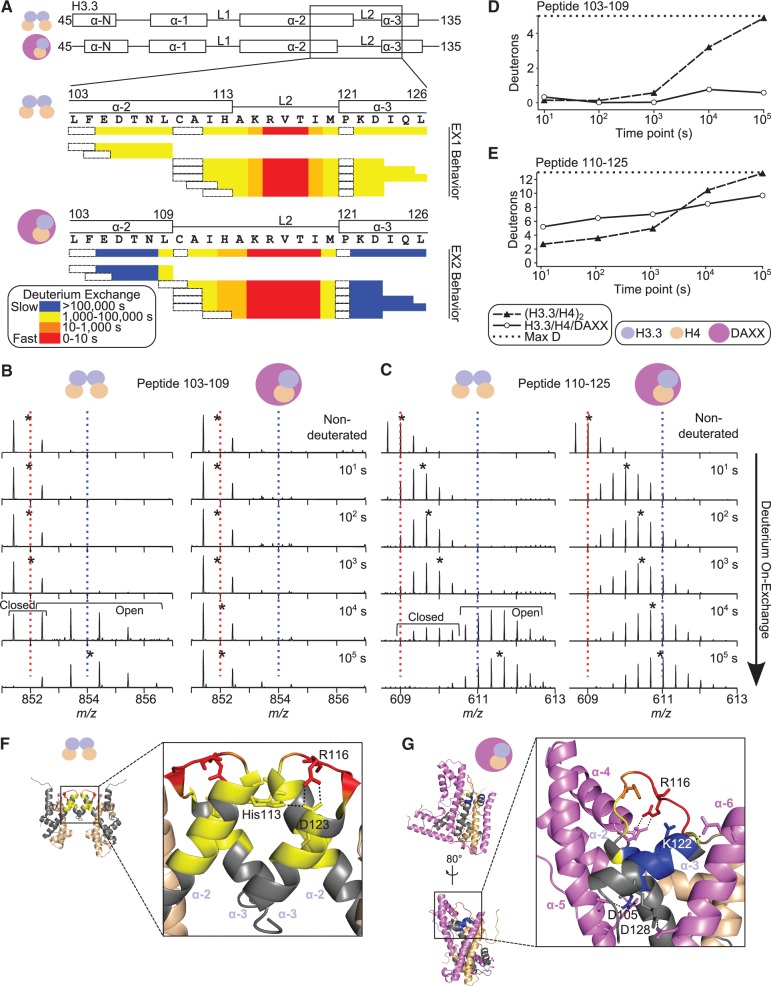
DAXX induces alterations to and prevents unfolding of the H3.3 α2-L2-α3 region in solution. (**A**) Deuterium exchange rate profile maps of peptides spanning H3.3 residues 103–126 in (H3.3/H4)_2_ (top) and H3.3/H4/DAXX (bottom). Schematics of the secondary structural features from the crystal structures of both protein complexes (Figure 1B and C, right) are shown, with the region of interest boxed and expanded below. The primary sequence and consensus exchange rate at each position are also shown. The first two residues of each peptide and prolines are boxed in dashed black lines because exchange of the first two backbone amide protons cannot be measured (30) and prolines lack amide protons. (**B** and **C**) MS data of two representative peptides, which are displayed as in Figure 2C. Both peptides from the (H3.3/H4)_2_ complex exhibit EX1 behavior (**D** and **E**). Comparison of H/DX for the indicated H3.3 peptides from each of the complexes. When data are biphasic, the reported number of deuterons is calculated from the average centroid value over the relative intensities of both the ‘open’ and ‘closed’ populations. The consensus exchange rates assigned in panel A are mapped onto either the (H3.3/H4)_2_ (PDB 3AV2) (**F**) or H3.3/H4/DAXX (PDB 4H9N) (**G**) crystal structures. Other portions of H3.3 are shown in gray. (F) H3.3 residues involved in the H3.3:H3.3′ four-helix bundle of (H3.3/H4)_2_ are shown. (G) H3.3 residues that establish contacts between H3.3 and DAXX in H3.3/H4/DAXX are highlighted.

As the only exception to the global stabilization of H3.3/H4 in the DAXX complex, an increase in H/DX in the H3.3 α2-L2-α3 region (Figure 1G and Supplementary Figure S2) indicates a local unfolding event. In the H3.3/H4/DAXX crystal structures, H3.3 L2 is extended at the expense of a full helical turn at the C-terminus of the H3.3 α2 helix (15,16). Using an H/DX rate-mapping strategy that has proven especially informative in other proteins/complexes with available high-resolution structures (19,29), we found that the reduced H/DX protection maps precisely to L2 of H3.3, which extends into the α2 helix in the DAXX complex (Figure 3A and G). In contrast, helical residues immediately adjacent to the extended loop, 105–108 and 122–126, are stabilized by 10-1000-fold upon DAXX binding (Figure 3A–E). From the co-crystal structure, specific salt bridges can be identified that stabilize the H3.3 α2-L2-α3 region in its extended conformation: DAXX residues D285 and D288 contact H3.3 R116 in L2; DAXX residues Q325 and R328, Q383 and N373 contact H3.3 D105, K122 and R128, respectively, in the helices (Figure 3G). These findings support the notion that the extended conformation of L2 observed in the complex crystal structure is also heavily populated in solution, with the H3.3 α2-L2-α3 region being strongly rigidified by DAXX as compared with the same region in the context of the (H3.3/H4)_2_ heterotetramer.

### DAXX prevents spontaneous, rapid unfolding of the H3.3 histone fold

We further investigated the nature of how DAXX globally rigidifies H3.3. In the (H3.3/H4)_2_ heterotetramer, all peptides spanning residues 103-126 display biphasic exchange behavior (i.e. EX1 or EX1-like kinetics). Under our reaction conditions, biphasic H/DX behavior is apparent at the 10^3^ s time point and most prominent at the 10^4^ s time point (Figure 3B and C). Although the *m/z* separation of the two biphasic populations is not as pronounced for short charge state +1 peptides (such as peptide 103–109 in Figure 3B), the exchange profile is distinct from that of EX2 exchanging peptides (Supplementary Figure S1C). EX1 or EX1-like behavior for many of the helical residues of H3.3 indicates that exchange occurs in an all-or-none manner, wherein after initial local helical unfolding, subsequent refolding is slower than the chemical H/DX rate (31).

Previous studies have confirmed the assumption that the H3/H4 dimers are in equilibrium with tetramers, heavily populating the dimer state at physiological salt concentrations (150 mM) (22,32). At 500 mM salt, at which our experiments were conducted, they more heavily populate a tetramer state. It is likely that the local unfolding and slow refolding (including residues 103–126) that we measure (Figure 3B and C) is linked to the loss of stabilizing H3.3:H3.3′ 4-helix bundle contacts that accompany transient loss of tetramerization (Figure 3F). Further, the timescale of these changes (i.e. EX1-like behavior is observed at 10^4^ s; Figure 3B and C) indicates slow interconversion between tetramer and dimer under the tested conditions.

In contrast to the (H3.3/H4)_2_ heterotetramer, the H3.3/H4 dimer bound by DAXX is completely protected from EX1 behavior. Peptide 103–109 of H3.3, for example, is nearly completely protected from H/DX, including EX1 exchange of the α2 helical residues (Figure 3B and D). Peptide 110–125 of H3.3 is not only representative of several peptides that show accelerated H/DX at initial time points upon DAXX binding because of the shortening of the α2 helix, but also shows protection at later time points (Figure 3C and E). This includes protection from the EX1 (or EX1-like) behavior of the α3 helical residues observed in the (H3.3/H4)_2_ heterotetramer. Thus, DAXX binding completely prevents the rapid spontaneous unfolding events observed within the H3.3/H4 histone fold, acting as a *bona fide* folding chaperone.

### DAXX completely refolds a partially unfolded dimer mutant version of H3.3/H4

**Figure 4.**
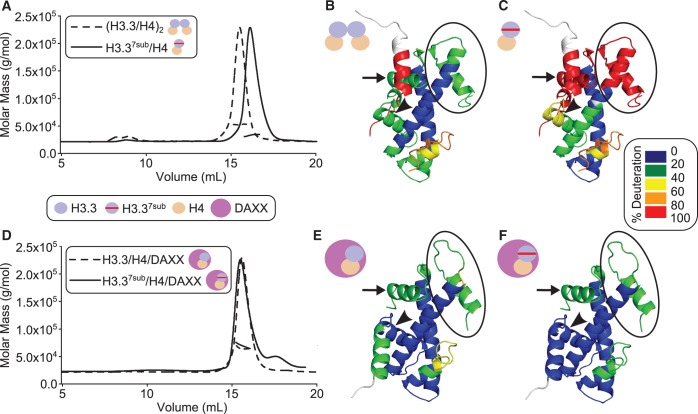
DAXX completely rescues the fold of a mutant version of H3.3/H4. SEC coupled with MALS and H/DX-MS of wild-type and mutant complexes of H3.3/H4 both without (**A–C**) and in complex with (**D–F**) DAXX. (A) The size of (H3.3/H4)_2_ is 53 kDa and that of H3.3^7sub^/H4 is 38 kDa. (D) The size of H3.3/H4/DAXX is 61 kDa and that of H3.3^7sub^/H4/DAXX is 58 kDa. (B, C, E and F) The consensus levels of H/DX at 10^1^ s for (H3.3/H4)_2_ (B), H3.3^7sub^/H4 (C), H3.3/H4/DAXX (E) and H3.3^7sub^/H4/DAXX (F) are mapped onto an H3.3/H4 dimer from either the H3.3 nucleosome (PDB 3AV2) (B and C) or H3.3^7sub^/H4/DAXX complex [PDB 4H9S; (15)] (E and F) crystal structures. Regions of H3.3 and H4 that are destabilized in H3.3^7sub^/H4 but are then stabilized when in complex with DAXX are highlighted as follows: the H3.3:H3.3′ tetramerization region is circled, and the H3.3 and H4 α1 helices are indicated by an arrowhead and arrow, respectively.

To further test the ability of DAXX to act as a molecular chaperone, we used a mutant version of the H3.3/H4 substrate (H3.3^7sub^/H4; Figure 4) with a substantial portion of the histone fold domain existing as an unfolded protein. The initial crystallographic forms of H3.3/H4/DAXX exploited a mutant of H3.3 with 5 or 7 substitutions aimed to rigidify the H3.3:H4 interface, essentially creating a H3.3/CENP-A hybrid [PDB 4H9N, PDB 4H9S; (15)]. While the mutant crystal structure is essentially identical with that of wild-type H3.3/H4/DAXX (16), we observed an unexpected consequence of introducing seven mutations in H3.3. The H3.3:H3.3′ interface is disrupted in the absence of DAXX, yielding a constitutive H3.3^7sub^/H4 dimer (Figure 4A). The H3.3^7sub^/H4 dimer has greatly extended regions of extremely rapid exchange (i.e. almost complete H/DX [>80%]) in both the H3.3:H3.3’ tetramerization four-helix bundle region as well as in the adjacent α1 helix of H4 (Figure 4B and C and Supplementary Figure S3). The H3.3 α1 helix is also more flexible in the H3.3^7sub^/H4 dimer than in the wild-type (H3.3/H4)_2_ heterotetramer (Figure 4B and C and Supplementary Figure S3) but shows some protection, suggesting that it is folded but samples unfolded states more frequently than its wild-type counterpart. Upon heterotrimer formation with DAXX (Figure 4D), however, nearly identical stability, as measured by its H/DX profile, is achieved in H3.3^7sub^/H4 as in its wild-type counterpart (Figure 4E and F). This stability includes all regions contacting DAXX and corroborates the notion that the protection we measure on H3.3/H4 upon DAXX binding is a consequence of preventing spontaneous unfolding events intrinsic to the histone fold.

### DAXX adopts a stable fold only upon binding H3.3/H4

The substantial stability conferred to H3.3/H4 by DAXX led us to investigate the nature of the DAXX:H3.3/H4 interfaces. We systematically analyzed contact points between DAXX and histones using PDBePISA (33) and found that they have an overall unusually hydrophobic character (Supplementary Figure S4). Such ‘dry’ interfaces are characteristic of a coupled binding-folding process, whereas polar contacts are more typical of binding between two fully folded monomers (34). Considering how DAXX might engage the histone complex, one could envision a series of hinge movements/rotations of well-folded DAXX helices that dock and sequentially envelop the histones. In this case, one would expect to observe measurable stability in the seven helices of the DAXX monomer with probable alterations in the linker regions, including contacts with histones and short secondary structural elements, which would likely be rearranged upon binding to histones. Alternatively, because the histones have many unfolded (or very rapidly unfolding/refolding) regions, DAXX might couple its own folding to engaging H3.3/H4 and exist in some disordered state in its free state. In this case, one would expect some or all of the DAXX helices to exhibit rapid H/DX, consistent with existing in an unfolded state or rapidly sampling unfolded conformations.

**Figure 5.**
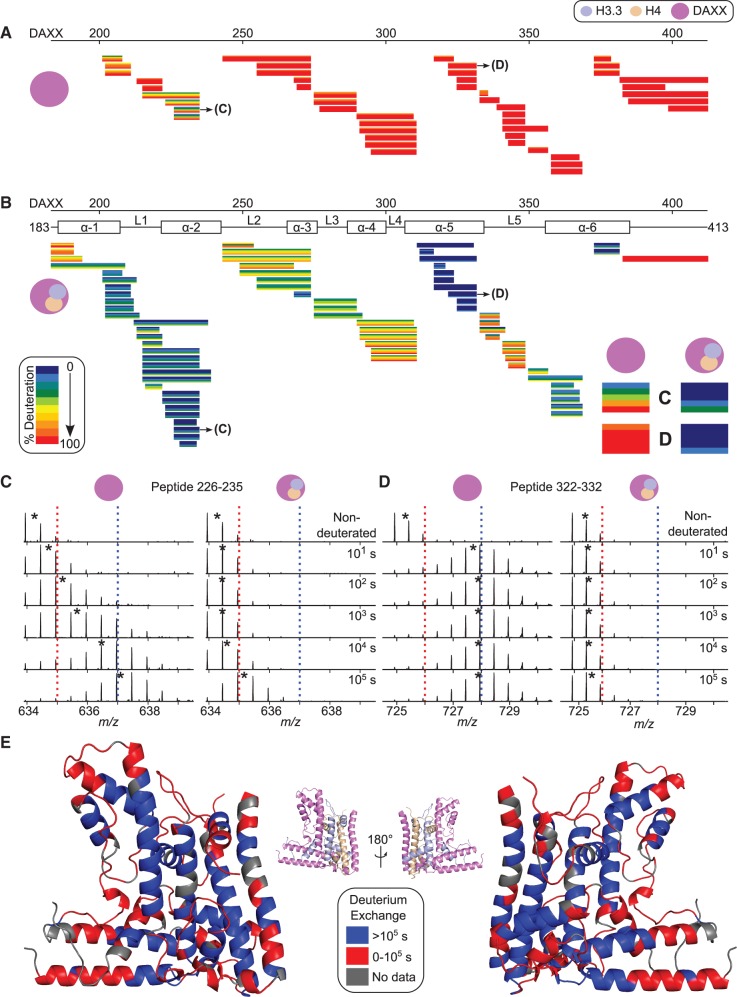
DAXX behaves essentially as an unfolded protein before binding H3.3/H4. H/DX data of peptides from the DAXX monomer (**A**) and in a heterotrimer complex with H3.3/H4 (**B**). Data are presented as in Figure 1B and C. Peptides spanning residues 226–235 (**C**) and 322–332 (**D**) are enlarged in the bottom right corner of panel B and their MS peptide spectra are shown, which are presented as in Figures 2 and 3. (**E**) The consensus exchange rate of each residue from the H3.3/H4/DAXX complex is mapped onto the crystal structure. Residues lacking any peptide coverage are colored gray.

To distinguish between these possibilities, we measured and compared the H/DX behavior of DAXX, either as a free monomer or bound to H3.3/H4 (Figure 5). Our analysis includes 84% coverage of the DAXX monomer (Figure 5A) and 94% coverage of DAXX from the heterotrimer complex (Figure 5B). Nearly complete exchange of the DAXX monomer occurs by the 10^1^ s or 10^2^ s time points in the vast majority of the protein (Figure 5A). In the DAXX ‘tower’ region (α1 and α2 helices), however, there is slower H/DX (representative peptide shown in Figure 5C), indicating that this region has a relatively stable fold before engaging histones. Outside of the ‘tower’ region, however, the DAXX monomer is essentially behaving as an unfolded protein. Upon binding to the histones, the stability of DAXX is vastly increased (Figure 5B). For example, peptide 322–332, which spans the α5 helix in the crystal structure, completely exchanges by 10^2^ s in unbound DAXX and has only just started to exchange by 10^5^ s when bound to the histones (Figure 5D). Thus, DAXX is stabilized to an even a greater extent than its own substrate, H3.3/H4.

Since we observed broad and substantial H/DX protection in all three chains of the H3.3/H4/DAXX heterotrimer, we investigated the slowest exchanging regions and their spatial arrangement in the complex (Figure 5E and Supplementary Figure S5). These very slow exchanging regions are found on each sub-unit (Figure 5E blue residues), especially on helices where there are close contacts with helices from the other two subunits. Thus, the H3.3/H4/DAXX heterotrimer is globally well folded, forming an extended hydrophobic core with equivalent H/DX protection on all major contact surfaces.

### H3.3 specific residues drive molecular recognition of DAXX by locking in stable structure

**Figure 6.**
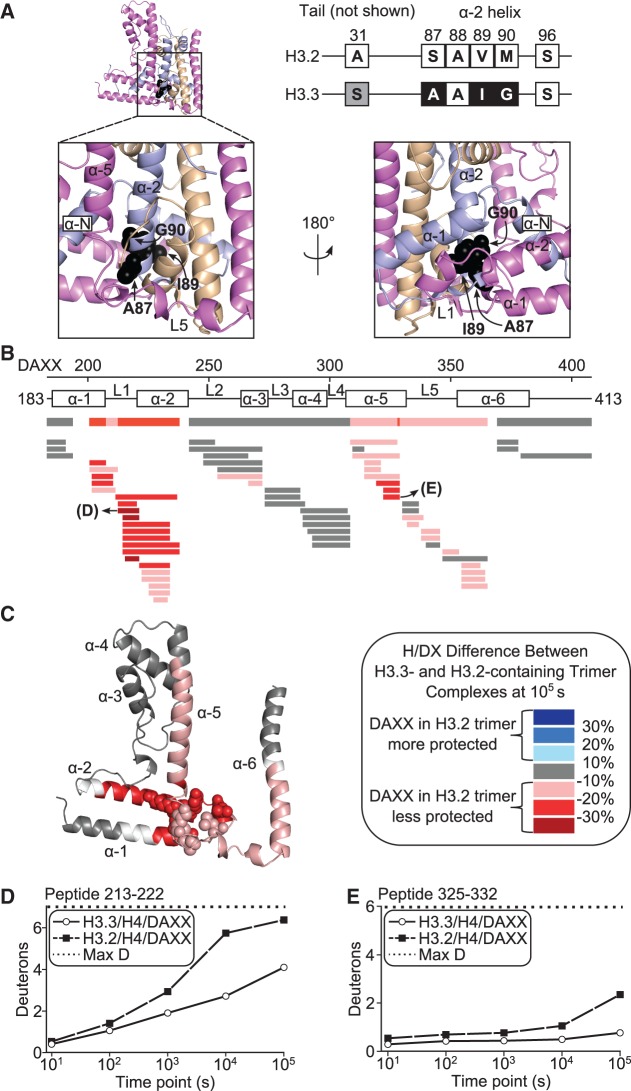
Stability induced in DAXX by contacts with H3.3-specific residues. (**A**) Differences in primary sequence between H3.2 and H3.3 histone variants. H3.3 residue S31 differs from H3.2 but is not important for recognition by DAXX. In contrast, H3.3 residues A87, I89 and G90 differ from H3.2 and are important for specific recognition by DAXX (8). These residues are shown in black space fill in the H3.3/H4/DAXX crystal structure (PDB 4H9N) to highlight the surrounding environment. (**B**) Decreased protection from H/DX of DAXX in complex with H3.2- compared with the H3.3-containing heterotrimer complex at 10^5^ s. The level of protection is determined by subtracting the percent deuteration of H3.2/H4/DAXX from that of H3.3/H4/DAXX for individual DAXX peptides, which are colored according to the legend. Gray represents no difference in H/DX between the two complexes, and white indicates the small number of positions lacking peptide coverage. Overlapping peptides at each position are assigned a consensus behavior, which is shown above the peptides. (**C**) The consensus difference at each residue is mapped onto DAXX from the H3.3/H4/DAXX crystal structure (PDB 4H9N). DAXX residues that line the area surrounding the H3.3-specificity residues are shown in space fill. (**D**) Comparison of H/DX for a DAXX peptide spanning L1. (**E**) Comparison of H/DX for a DAXX peptide spanning the α5 helix.

A key unresolved issue is the mechanism by which DAXX specifically associates with H3.3/H4 despite a high cellular concentration of nearly identical canonical H3 (H3.1 and H3.2). Specificity for H3.3 is clearly observed using purified components in binding assays (8,9,15,16). Biochemical and structural data have been used to propose that G90, and to a lesser extent A87, of H3.3, which exist in a polar cavity and shallow hydrophobic pocket, respectively, are the principal determinants for DAXX recognition *in vivo* and *in vitro* (15,16) (the location of these residues in the heterotrimer complex is shown in Figure 6A). Importantly, the ‘tower’ helices alone were able to discriminate H3.3 from H3.2 based on the side chain of M90 in H3.2 (15). Along with our new finding that monomeric DAXX is unfolded, this might suggest a ‘lock-and-key’ mechanism, where the folding of DAXX generates an interface that only accommodates the lack of a side chain on H3.3 G90. However, a co-crystal structure with H3.3 G90M shows that the substantial side chain of methionine can be accommodated in a hydrophilic cavity with minimal conformational deviations in backbone and side chains throughout DAXX (15). Therefore, discrimination does not rely on direct steric exclusion, arguing against a simple ‘lock-and-key’ recognition mechanism.

The relatively small surface area surrounding the recognition determinants (131 Å^2^ [=buried surface area of A87, I89 and G90 in H3.3/H4/DAXX complex], PDBePISA) is in stark contrast to the total buried surface area of H3.3/H4 in the entire complex (4500 Å^2^) (15). Thus, on enveloping either H3.1/H4, H3.2/H4 or H3.3/H4, the vast majority of the interaction interface is identical, making the finding that DAXX selectively associates with H3.3/H4 (8,9), and not H3.1/H4 or H3.2/H4, so remarkable. To address dynamic differences in molecular recognition of histone variant complexes by DAXX, we reconstituted the H3.2/H4/DAXX heterotrimer, performed H/DX-MS (Figure 1A) and compared the DAXX subunit with that when bound to H3.3/H4 (Figure 6 and Supplementary Figures S6–S9). We found that DAXX adopts a similar conformation when bound to H3.2 as when bound to H3.3, sharing the same general fast and slow exchanging regions (Supplementary Figure S6). At many locations, DAXX displays essentially identical H/DX rates in either H3.2- or H3.3-containing complexes (Figures 6B and C and Supplementary Figures S7 gray peptides/residues and S8). Importantly though, there is a broad region of flexibility found in DAXX near the contact region with the histone recognition residues when it binds to H3.2 rather than H3.3 (Figure 6C). Specifically, less protection is most apparent in the DAXX ‘tower’ region, including L1, and also in the region surrounding L5 from the α5 to α6 helices when inappropriately bound to H3.2/H4 instead of its natural substrate, H3.3/H4 (Figure 6B and C and Supplementary Figure S7). The regions of DAXX that are clearly stabilized by H3.3 specific contacts (Figure 6B and C) overlap with the most stably folded portions of DAXX in the heterotrimer complex (Figure 5B).

Evident in the crystal structure of the H3.3/H4/DAXX complex, DAXX L1 is highly ordered: residues 212–216 form a 3(10) helix and nearby residues 209–210 interact in a β sheet-like manner with residues 84–85 of H3.3 (15). The protection from exchange in this region in the H3.3/H4/DAXX complex, particularly spanning the 3(10) helix (representative peptide shown in Figure 6D), attests to the minimal dynamics/conformational flexibility in this region. For peptides spanning DAXX residues Glu209 and Leu210, the protection we observe must be due to the inter-chain hydrogen bonding with H3.3, involving the amide protons at these positions interacting with residues Arg84 and Phe85 from H3.3 (15). Thus, it is one of the only regions in which our H/DX data correlate directly to protection of amide protons through direct H-bonding between different polypeptides. As is the case for most other protein–protein interactions that have been studied, however, the bulk of H/DX protection arises from stabilization of structural elements involving intra-chain H-bonding (e.g. H-bonding within α-helices or between β-strands). The increase in H/DX in the corresponding H3.2-containing complex suggests that the H3.3-specific residues are critical for stabilizing the fold of the 3(10) helix in DAXX L1 (Figure 6D and Supplementary Figure S9), as well as the C-terminal portion of the DAXX α5 helix (Figure 6E and Supplementary Figure S9). It takes these peptides 10–50 times longer to achieve the same level of deuteration in H3.3 relative to H3.2 (Figure 6D and E). Therefore, the selective binding of H3.3 is mediated by the stable fold in the helices surrounding the H3.3-specific residues. The imperfect fit with H3.2 and the resulting destabilization of DAXX local secondary structure provide a protein folding-based rationale for decreased binding relative to its *bona fide* substrate, H3.3.

## DISCUSSION

Using H/DX-MS, we define the major protein folding implications of forming the H3.3/H4/DAXX complex. Our solution data confirm key features and interactions derived from crystallographic data of the complex and, more importantly, yield novel implications for the chaperone-specific assembly/disassembly of the complex. Almost completely disordered in its free state, DAXX folding is coupled to binding a H3.3/H4 dimer (Figure 5). Vice versa, DAXX is capable of rigidifying transiently unfolding regions in the histone fold (e.g. the α2 and α3 helices of H3.3; Figure 3) and folding an otherwise unfolded (or extremely rapidly unfolding) region (e.g. the αN helix of H3.3; Figure 2). Moreover, DAXX is able to fold a mutant version of H3.3 where a substantial portion of the histone fold domain is initially unfolded (Figure 4). Importantly, replacing H3.3 with a non-cognate variant H3.2 in the complex leads to a decreased stability of local secondary structure elements of DAXX (Figure 6).

### A model for coupled binding–folding of DAXX and histones H3.3/H4

Combining our HD/X data of free and bound histones and DAXX chaperone, we are able to propose a model for the coupled binding-folding of DAXX with histones H3.3/H4. In this model, a mostly unfolded DAXX makes initial contacts near the H3.3 specificity region and can then sample a large part of the histone surface before folding into place. Partially folded intermediates might play a crucial role in guiding the formation of the proper complex, in which case their dynamic stability would be responsible for the discrimination of H3.3 from other H3 variants.

Residual secondary structure in the ‘tower’ helices (Figure 5A) suggests that initial ordered contacts with histones H3.3/H4 could be made through a pre-folded ‘tower’ rather than other, completely unstructured parts of DAXX. Based on the finding that DAXX experiences local instability in the ‘tower’ helices and surrounding local environment when bound to H3.2 (Figure 6), we propose that the H3 variant residues in the H3.3 α2 helix have an energetic impact on the folding step(s) immediately following initial contact in complex assembly. In support, previous biochemical data show that the DAXX ‘tower’ alone binds H3.3/H4 tightly but does not interact with canonical H3.1/2 (15).

**Figure 7.**
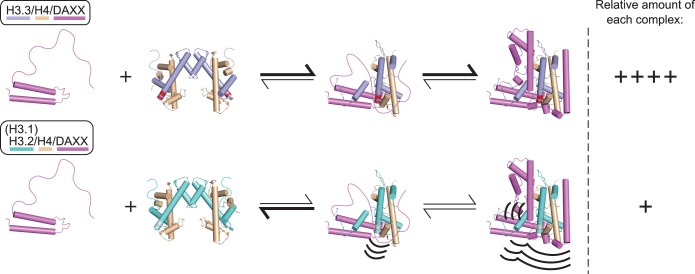
A stepwise co-folding model to explain how H3.3-specific assembly with DAXX is achieved. See text for details.

We propose a working model in which the L1 of the DAXX ‘tower’ first guides the association, as it contains an acidic (Glu-rich) region that can make long-range electrostatic interactions with the highly basic lateral histone H3.3 surface. Through their compatible hydrophobic surfaces, the ‘tower’ helices, including portions of the intervening loop, could subsequently fully fold onto the H3.3 α1 helix, L1 and α2 helix. The tight wrapping of DAXX L2 around the H3.3 αN helix seen in the co-structures of H3.3/H4/DAXX indicates that prior folding of the αN helix of H3.3 is first required in order for the fully folded DAXX complex to form (Figure 1C) because it seems sterically unlikely it would fold after DAXX has folded. The H/DX data indicate that the αN helix is unstructured in free heterotetramer but stable in the DAXX complex (Figure 2). Thus, it seems likely that binding of the DAXX ‘tower’ captures the unstructured N-terminus of H3.3 and directs folding of the H3.3 αN helix (Figure 7).

Throughout this sequence of folding steps, the cognate H3.3-specific residues in the α2 helix might play an important role in maintaining stable association of DAXX with the histones, whereas non-cognate residues in H3.2 might destabilize the interface and promote dissociation of DAXX before it is fully folded (Figure 7, middle; instability of H3.2-containing complex represented by curved lines near the regions destabilized in DAXX). We propose that only after these initial associations and folding steps, the remaining DAXX structural elements can form, with the long L2 threading across the H3.3 αN helix and the α5 and α6 helices packing against H3.3 and H4, respectively (Figure 7, right). The pronounced stability of the H3.3/H4/DAXX heterotrimer is confounded by the large buried interface area gained on these last folding steps in our model. While we observe local destabilization of DAXX elements in a non-cognate H3.2/H4/DAXX complex (within the ‘tower’ and the helices surrounding L5, see Figure 6), these perturbations are not large enough to efficiently disassemble a fully folded complex. Our model, therefore, provides a rationale for how high selectivity and high affinity can be achieved by a sequential folding pathway: one or more energetic barriers imposed early in the folding pathway can disfavor the formation of the final high-affinity complex with a non-cognate histone H3 variant. Likewise, in the presence of competing histone chaperones [such as CAF1, ASF1 (1)] and assembly reactions, a small energetic barrier imposed by non-cognate histone H3.1/2 residues might be sufficient to disfavor inappropriate assembly with DAXX. Future experiments to further test the working model we propose is likely to yield important additional insight into the DAXX chromatin assembly pathway.

Coupled binding-folding of protein interactions, such as we find for H3.3/H4/DAXX, has been hypothesized to enhance molecular recognition specificity (35). Our data argue strongly that selectivity for H3.3 versus H3.2 must occur early, before the completion of co-folding of an inappropriate H3.2/H4/DAXX heterotrimer that, once formed, would be unlikely to rapidly dissociate given the extensive and intertwined interfaces between DAXX and the histones. In addition to variant-specific residues, post-translational modifications, such as K56 acetylation and methylation (36–38) in the H3.3 αN helix, might affect proper folding of the DAXX complex. Predominantly recognized for their function within the nucleosomal context, such modifications also affect chaperone interactions (39). However, there is no evidence to date that H3 variants are modified differentially to enhance recognition specificity. In particular, the heavily modified H3 N-terminal tail does not seem to be recognized by DAXX (15), making a regulatory function of these modifications in complex assembly unlikely.

Alternative to our proposed model, the complex could be folded through (i) the simultaneous collapse of all DAXX structural elements onto a H3.3/H4 dimer with a transiently ordered αN helix or (ii) the reverse folding sequence with α5 and α6 helices making the initial contacts. In disagreement with the first alternative scenario and consistent with previous studies (18,40), we found no evidence for the formation of a αN helix on free H3.3/H4. The reversed folding order, where DAXX α5 and α6 helices fold first onto the H3.3/H4 dimer, is plausible. However, complex formation would be limited by the availability of ‘free’ H3.3/H4 dimers *in vivo*, as contacts in this region are incompatible with a (H3.3/H4)_2_ heterotetramer or H3.3/H4/ASF1 heterotrimer complex (15). In addition, our H/DX data indicate that the H3.3 α2 helix is partially unwound in solution when contacting the α5 and α6 helices of DAXX (Figure 3). Without cooperative binding contributions from the rest of DAXX, initial association of DAXX α5/α6 helices with the H3.3 homodimerization interface, therefore, seems likely to be thermodynamically disfavored.

### Disassembly of H3.3/H4/DAXX, nucleosome assembly and the implications of cooperative folding/unfolding

Mirroring the coupled binding-folding, our data suggest that disassembly of the H3.3/H4/DAXX complex would require widespread unfolding of DAXX. It remains to be seen if disassembly of the complex is regulated by DNA, for example, in a way where H3.3/H4 dimers are handed onto DNA in a concerted mechanism. In such a model, histone chaperones might stabilize certain structures necessary for efficient deposition onto chromatin that are distinct from the final nucleosomal arrangement. We speculate, for example, that in the context of DNA or another chaperone, a second H3.3/H4 dimer could compete with DAXX for the H3.3 homodimerization interface, reversing the local unwinding of the α2 helix and ejecting DAXX. *In vitro* experiments suggest that ASF1 is capable of taking over the H3.3/H4 dimer from a DAXX complex (15), raising the possibility of a multi-step disassembly reaction that warrants future investigation.

The histone chaperone DAXX has emerged as a pivotal example of a family of histone chaperones, also including HJURP (4,5,17), Scm3 (41) and Chz1 (42), that bind specific histone variants with high specificity. For HJURP and Scm3, there appears to be a hydrophobic effect-driven binding (or coupled binding-folding) as opposed to a DNA-mimicking electrostatic attraction (43). In addition, some of the dynamic features of the H3.3/H4/DAXX complex are highly reminiscent of a previously characterized CENP-A/H4/HJURP complex (17), and rely on similar structural elements (44). However, the nature of DAXX to envelop its histone substrates (15,16) is exceptional among a limited number of histone chaperone complexes that have been examined with high-resolution structural and/or dynamic approaches. A recent study of Nap1, for example, showed that the chaperone is well folded before encountering H2A/H2B dimers (20), but it remains to be seen, which among the spectrum of other histone chaperones co-fold with their substrates. Importantly, HJURP (17), Nap1 (20) and DAXX highlight a previously unrecognized feature that might be common to many, if not all, histone chaperones: the promotion of a folded histone state. Such function might be crucial to avoid spontaneous unfolding and aggregation in the cellular environment on the pathway to assembling H3.3-containing nucleosomes.

Our study further highlights the surprisingly large extent to which reversible folding of whole protein domains can depend on as an appropriate protein partner. Intrinsically unstructured regions in proteins have long been recognized to be able to adopt defined structures with their interaction partners and to provide adaptable platforms for protein–protein interactions (45). However, interaction motifs described so far are limited to relatively short linear motifs with random-coil characteristics (such as an unusually high ratio of charged to hydrophobic residues). Chz1, for example, has an intrinsically disordered region, a small portion of which adopts stable secondary structure upon binding the H2A.Z/H2B histone dimer (46). In contrast, DAXX exhibits stereotypical primary sequence features of a *bona fide* globular folded domain. Our direct structural and dynamic insight is instrumental in understanding the folding of DAXX, raising the possibility that domain-level cooperative folding is an underappreciated feature of protein–protein interactions. We envision that similarly complex protein–protein interactions exist in diverse biological contexts beyond histone chaperones, contributing to both molecular recognition and affinity.

## SUPPLEMENTARY DATA

Supplementary Data are available at NAR Online.

## FUNDING

National Institutes of Health [GM082989 to B.E.B.]; a Career Award in the Biomedical Sciences from the Burroughs Wellcome Fund (to B.E.B.); a Rita Allen Foundation Scholar Award (to B.E.B.); and an EMBO Long Term Fellowship [ALTF 1232-2011 to S.J.E.]. Funding for open access charge: NIH research grant.

*Conflict of interest statement*. None declared.

## Supplementary Material

Supplementary Data

## References

[1] Hondele M, Ladurner AG (2011). The chaperone-histone partnership: for the greater good of histone traffic and chromatin plasticity. Curr. Opin. Struct. Biol..

[2] Ray-Gallet D, Quivy J-P, Scamps C, Martini EM-D, Lipinski M, Almouzni G (2002). HIRA is critical for a nucleosome assembly pathway independent of DNA synthesis. Mol. Cell.

[3] Tagami H, Ray-Gallet D, Almouzni G, Nakatani Y (2004). Histone H3.1 and H3.3 complexes mediate nucleosome assembly pathways dependent or independent of DNA synthesis. Cell.

[4] Foltz DR, Jansen LET, Bailey AO, Yates JR, Bassett EA, Wood S, Black BE, Cleveland DW (2009). Centromere-specific assembly of CENP-A nucleosomes is mediated by HJURP. Cell.

[5] Dunleavy EM, Roche D, Tagami H, Lacoste N, Ray-Gallet D, Nakamura Y, Daigo Y, Nakatani Y, Almouzni-Pettinotti G (2009). HJURP is a cell-cycle-dependent maintenance and deposition factor of CENP-A at centromeres. Cell.

[6] Shuaib M, Ouararhni K, Dimitrov S, Hamiche A (2010). HJURP binds CENP-A via a highly conserved N-terminal domain and mediates its deposition at centromeres. Proc. Natl Acad. Sci. USA.

[7] Goldberg AD, Banaszynski LA, Noh K-M, Lewis PW, Elsaesser SJ, Stadler S, Dewell S, Law M, Guo X, Li X (2010). Distinct factors control histone variant H3.3 localization at specific genomic regions. Cell.

[8] Lewis PW, Elsaesser SJ, Noh K-M, Stadler SC, Allis CD (2010). Daxx is an H3.3-specific histone chaperone and cooperates with ATRX in replication-independent chromatin assembly at telomeres. Proc. Natl Acad. Sci. USA.

[9] Drané P, Ouararhni K, Depaux A, Shuaib M, Hamiche A (2010). The death-associated protein DAXX is a novel histone chaperone involved in the replication-independent deposition of H3.3. Genes Dev..

[10] Verreault A, Kaufman PD, Kobayashi R, Stillman B (1996). Nucleosome assembly by a complex of CAF-1 and acetylated histones H3/H4. Cell.

[11] Jiao Y, Shi C, Edil BH, de Wilde RF, Klimstra DS, Maitra A, Schulick RD, Tang LH, Wolfgang CL, Choti MA (2011). DAXX/ATRX, MEN1, and mTOR pathway genes are frequently altered in pancreatic neuroendocrine tumors. Science.

[12] Schwartzentruber J, Korshunov A, Liu X-Y, Jones DTW, Pfaff E, Jacob K, Sturm D, Fontebasso AM, Quang D-AK, Tönjes M (2012). Driver mutations in histone H3.3 and chromatin remodelling genes in paediatric glioblastoma. Nature.

[13] Heaphy CM, de Wilde RF, Jiao Y, Klein AP, Edil BH, Shi C, Bettegowda C, Rodriguez FJ, Eberhart CG, Hebbar S (2011). Altered telomeres in tumors with ATRX and DAXX mutations. Science.

[14] Escobar-Cabrera E, Lau DKW, Giovinazzi S, Ishov AM, Mcintosh LP (2010). Structural characterization of the DAXX N-terminal helical bundle domain and its complex with Rassf1C. Structure.

[15] Elsaesser SJ, Huang H, Lewis PW, Chin JW, Allis CD, Patel DJ (2012). DAXX envelops an H3.3-H4 dimer for H3.3-specific recognition. Nature.

[16] Liu C-P, Xiong C, Wang M, Yu Z, Yang N, Chen P, Zhang Z, Li G, Xu R-M (2012). Structure of the variant histone H3.3–H4 heterodimer in complex with its chaperone DAXX. Nat. Struct. Mol. Biol..

[17] Bassett EA, DeNizio J, Barnhart-Dailey MC, Panchenko T, Sekulic N, Rogers DJ, Foltz DR, Black BE (2012). HJURP uses distinct CENP-A surfaces to recognize and to stabilize CENP-A/histone H4 for centromere assembly. Dev. Cell.

[18] Black BE, Foltz DR, Chakravarthy S, Luger K, Woods VL, Cleveland DW (2004). Structural determinants for generating centromeric chromatin. Nature.

[19] Panchenko T, Sorensen TC, Woodcock CL, Kan Z-Y, Wood S, Resch MG, Luger K, Englander SW, Hansen JC, Black BE (2011). Replacement of histone H3 with CENP-A directs global nucleosome array condensation and loosening of nucleosome superhelical termini. Proc. Natl Acad. Sci. USA.

[20] D'Arcy S, Martin KW, Panchenko T, Chen X, Bergeron S, Stargell LA, Black BE, Luger K (2013). Chaperone Nap1 shields histone surfaces used in a nucleosome and can put H2A-H2B in an unconventional tetrameric form. Mol. Cell.

[21] Englander SW (2006). Hydrogen exchange and mass spectrometry: A historical perspective. J. Am. Soc. Mass Spectrom..

[22] Donham DC, Scorgie JK, Churchill MEA (2011). The activity of the histone chaperone yeast Asf1 in the assembly and disassembly of histone H3/H4-DNA complexes. Nucleic Acids Res..

[23] Kan ZY, Mayne L, Chetty PS, Englander SW (2011). ExMS: data analysis for HX-MS experiments. J. Am. Soc. Mass Spectrom..

[24] Walters BT, Ricciuti A, Mayne L, Englander SW (2012). Minimizing back exchange in the hydrogen exchange-mass spectrometry experiment. J. Am. Soc. Mass Spectrom..

[25] Pantazatos D, Kim JS, Klock HE, Stevens RC, Wilson IA, Lesley SA, Woods VL (2004). Rapid refinement of crystallographic protein construct definition employing enhanced hydrogen/deuterium exchange MS. Proc. Natl Acad. Sci. USA.

[26] Luger K, Mäder AW, Richmond RK, Sargent DF, Richmond TJ (1997). Crystal structure of the nucleosome core particle at 2.8 A resolution. Nature.

[27] Tachiwana H, Osakabe A, Shiga T, Miya Y, Kimura H, Kurumizaka H (2011). Structures of human nucleosomes containing major histone H3 variants. Acta. Crystallogr., Sect. D. Biol. Crystallogr..

[28] Black BE, Brock MA, Bédard S, Woods VL, Cleveland DW (2007). An epigenetic mark generated by the incorporation of CENP-A into centromeric nucleosomes. Proc. Natl Acad. Sci. USA.

[29] Sekulic N, Bassett EA, Rogers DJ, Black BE (2010). The structure of (CENP-A-H4)(2) reveals physical features that mark centromeres. Nature.

[30] Bai Y, Milne JS, Mayne L, Englander SW (1993). Primary structure effects on peptide group hydrogen exchange. Proteins.

[31] Englander SW, Kallenbach NR (1983). Hydrogen exchange and structural dynamics of proteins and nucleic acids. Q. Rev. Biophys..

[32] Winkler DD, Zhou H, Dar MA, Zhang Z, Luger K (2012). Yeast CAF-1 assembles histone (H3-H4)2 tetramers prior to DNA deposition. Nucleic Acids Res..

[33] Krissinel E, Henrick K (2007). Inference of macromolecular assemblies from crystalline state. J. Mol. Biol..

[34] Levy Y, Onuchic JN (2006). Water mediation in protein folding and molecular recognition. Ann. Rev. Biophys. Biom..

[35] Dyson HJ, Wright PE (2005). Intrinsically unstructured proteins and their functions. Nat. Rev. Mol. Cell Biol..

[36] Xu F, Zhang K, Grunstein M (2005). Acetylation in histone H3 globular domain regulates gene expression in yeast. Cell.

[37] Recht J, Tsubota T, Tanny JC, Diaz RL, Berger JM, Zhang X, Garcia BA, Shabanowitz J, Burlingame AL, Hunt DF (2006). Histone chaperone Asf1 is required for histone H3 lysine 56 acetylation, a modification associated with S phase in mitosis and meiosis. Proc. Natl Acad. Sci. USA.

[38] Yu Y, Song C, Zhang Q, DiMaggio PA, Garcia BA, York A, Carey MF, Grunstein M (2012). Histone H3 lysine 56 methylation regulates DNA replication through its interaction with PCNA. Mol. Cell.

[39] Su D, Hu Q, Li Q, Thompson JR, Cui G, Fazly A, Davies BA, Botuyan MV, Zhang Z, Mer G (2012). Structural basis for recognition of H3K56-acetylated histone H3–H4 by the chaperone Rtt106. Nature.

[40] Bowman A, Ward R, El-Mkami H, Owen-Hughes T, Norman DG (2010). Probing the (H3-H4)2 histone tetramer structure using pulsed EPR spectroscopy combined with site-directed spin labelling. Nucleic Acids Res..

[41] Mizuguchi G, Xiao H, Wisniewski J, Smith MM, Wu C (2007). Nonhistone Scm3 and histones CenH3-H4 assemble the core of centromere-specific nucleosomes. Cell.

[42] Luk E, Vu N-D, Patteson K, Mizuguchi G, Wu W-H, Ranjan A, Backus J, Sen S, Lewis M, Bai Y (2007). Chz1, a nuclear chaperone for histone H2AZ. Mol. Cell.

[43] Elsaesser SJ, D'Arcy S (2012). Towards a mechanism for histone chaperones. Biochim. Biophys. Acta..

[44] Elsaesser SJ (2013). A common structural theme in histone chaperones mimics interhistone contacts. Trends Biochem. Sci..

[45] Babu MM, Kriwacki RW, Pappu RV (2012). Structural biology. Versatility from protein disorder. Science.

[46] Zhou Z, Feng H, Hansen DF, Kato H, Luk E, Freedberg DI, Kay LE, Wu C, Bai Y (2008). NMR structure of chaperone Chz1 complexed with histones H2A.Z-H2B. Nat. Struct. Mol. Biol..

