# Supplementary material for: DAXX co-folds with H3.3/H4 using high local stability conferred by the H3.3 variant recognition residues
Source: Nucleic Acids Res. 2014 Jan 31;42(7):4318–31. doi: 10.1093/nar/gku090 (PMC3985662; doi:10.1093/nar/gku090)
Supplement: Supplementary Data [file supp_42_7_4318__index.html]

DAXX co-folds with H3.3/H4 using high local stability conferred by the H3.3 variant recognition residues — DAXX co-folds with H3.3/H4 using high local stability conferred by the H3.3 variant recognition residues — Supplementary Data 

# DAXX co-folds with H3.3/H4 using high local stability conferred by the H3.3 variant recognition residues

## Supplementary Data

files

**Files in this Data Supplement:**

- Supplementary Data - pdf file
