# Supplementary material for: DAXX co-folds with H3.3/H4 using high local stability conferred by the H3.3 variant recognition residues
Source: Nucleic Acids Res. 2014 Jan 31;42(7):4318–31. doi: 10.1093/nar/gku090 (PMC3985662; doi:10.1093/nar/gku090)

**SUPPLEMENTARY MATERIAL**

**Supplemental Figure Legends**

**Supplemental Figure S1.** Stabilization of H3.3 and H4 upon DAXX binding. (**A**) The locations of a H3.3 peptide (residues 61-69) and a H4 peptide (residues 53-60) are shown in purple and yellow, respectively, on both the $(H3.3/H4)_2$ (PDB 3AV2) and H3.3/H4/DAXX (PDB 4H9N) crystal structures. Comparison of H/DX from both complexes over the time course and side-by-side analysis of MS data for the H3.3 peptide (**B**) and the H4 peptide (**C**) are shown. The data are displayed as in Figure 2. The dashed lines in panel C and D represent the maximum number of measurable deuterons for these peptides.

**Supplemental Figure S2.** Difference in H/DX of H3.3/H4 upon heterotrimer formation with DAXX. Peptide profiles of the H/DX difference of H3.3 and H4 between $(H3.3/H4)_2$ and H3.3/H4/DAXX at $10^1$ s, $10^2$ s, $10^3$ s, $10^4$ s, and $10^5$ s. The level of protection is determined by subtracting the percent deuteration of H3.3/H4/DAXX from that of $(H3.3/H4)_2$ for individual peptides. Data are displayed as in Figure 6 but are colored according to the legend. The consensus behavior for each residue of H3.3 and H4 at each time point is shown above the peptides and is mapped onto the crystal structure of H3.3/H4 from the heterotrimer complex (PDB 4H9N) to the right of the corresponding data. The region boxed by a solid line is discussed in detail in Figure 2 (H3.3 αN helix), and that bracketed by a dashed line is highlighted in Figure 3 (H3.3 α2-L2-α3 region).

**Supplemental Figure S3.** DAXX completely rescues an unstable dimer mutant of H3.3/H4. H/DX data at $10^1$ s for H3.3/H4 from $(H3.3/H4)_2$ (**A**), $H3.3^{7sub}$/H4 (**B**), H3.3/H4/DAXX (**C**), and $H3.3^{7sub}$/H4/DAXX (**D**). Each horizontal bar represents an individual peptide and is color coded for percent deuteration as indicated in the legend. The regions of H3.3 and H4 that are destabilized in $H3.3^{7sub}$/H4 but are then stabilized upon binding DAXX are boxed in dashed lines.

**Supplemental Figure S4.** Overall hydrophobic nature of the interface between DAXX and H3.3/H4. DAXX residues are colored on the H3.3/H4/DAXX crystal structure (PDB 4H9N) based on their solvation energy term of complex formation. Residues colored orange are those with positive solvation energy and represent hydrophobic contacts with H3.3/H4. Residues colored purple are those with negative solvation energy and represent polar contacts with H3.3/H4.

**Supplemental Figure S5.** Deuterium exchange rates of H3.3/H4/DAXX. Deuterium exchange rate profile maps of H3.3 (**A**), H4 (**B**), and DAXX (**C**) from the H3.3/H4/DAXX heterotrimer complex. The consensus exchange rate of each residue, which is mapped onto the crystal structure in Figure 5E, is shown above the peptides.

**Supplemental Figure S6.** Comparison of H/DX-MS of DAXX bound to H3.3/H4 (**A**, data reproduced from Figure 5B) and H3.2/H4 (**B**). Data are displayed as in Figures 1B,C and 5A,B.

**Supplemental Figure S7.** H3.2/H4 forms a less stable heterotrimer complex with DAXX than does H3.3/H4. Peptide profiles of the H/DX difference of DAXX between H3.3/H4/DAXX and H3.2/H4/DAXX at $10^1$ s, $10^2$ s, $10^3$ s, $10^4$ s, and $10^5$ s. The level of protection is determined by subtracting the percent deuteration of H3.2/H4/DAXX from that of H3.3/H4/DAXX for individual DAXX peptides. Data are displayed as in Figure 6 but are colored according to the legend.

**Supplemental Figure S8.** Regions of DAXX distal to the H3.3 specificity residues experience similar H/DX levels in both the H3.3- and H3.2-containing complexes. (**A**) The location of a DAXX peptide (residues 293-311) spanning the α4 to α5 helices is shown in black on the H3.3/H4/DAXX crystal structure (PDB 4H9N). (**B**) Comparison of H/DX for the peptide from both the H3.3-containing and H3.2-containing heterotrimer complexes over the time course. The maximum number of measurable deuterons is shown by a black dotted line. (**C**) Side-by-side analysis of MS data for the indicated peptide from the H3.3-containing complex (left) or the H3.2-containing complex (right). Data are displayed as in Figure 2.

**Supplemental Figure S9.** Regions of DAXX near H3.3-specificity residues show a consistent difference in H/DX between the H3.3- and H3.2-containing complexes. Replicate MS data from H3.3/H4/DAXX ($2^{nd}$ and $3^{rd}$ row spectra) and H3.2/H4/DAXX ($4^{th}$ and $5^{th}$ row spectra) at $10^5$s of DAXX peptides spanning residues 213-222 (**A**) and residues 325-332 (**B**), which are also displayed in Figure 6D and E, respectively. Data are displayed as in Figure 2, such that the stars denoting the centroid values and dotted guidepost-lines serve to highlight the significantly greater *m/z* shift of the peptides from the H3.2-containing complex compared to the H3.3-containing complex.

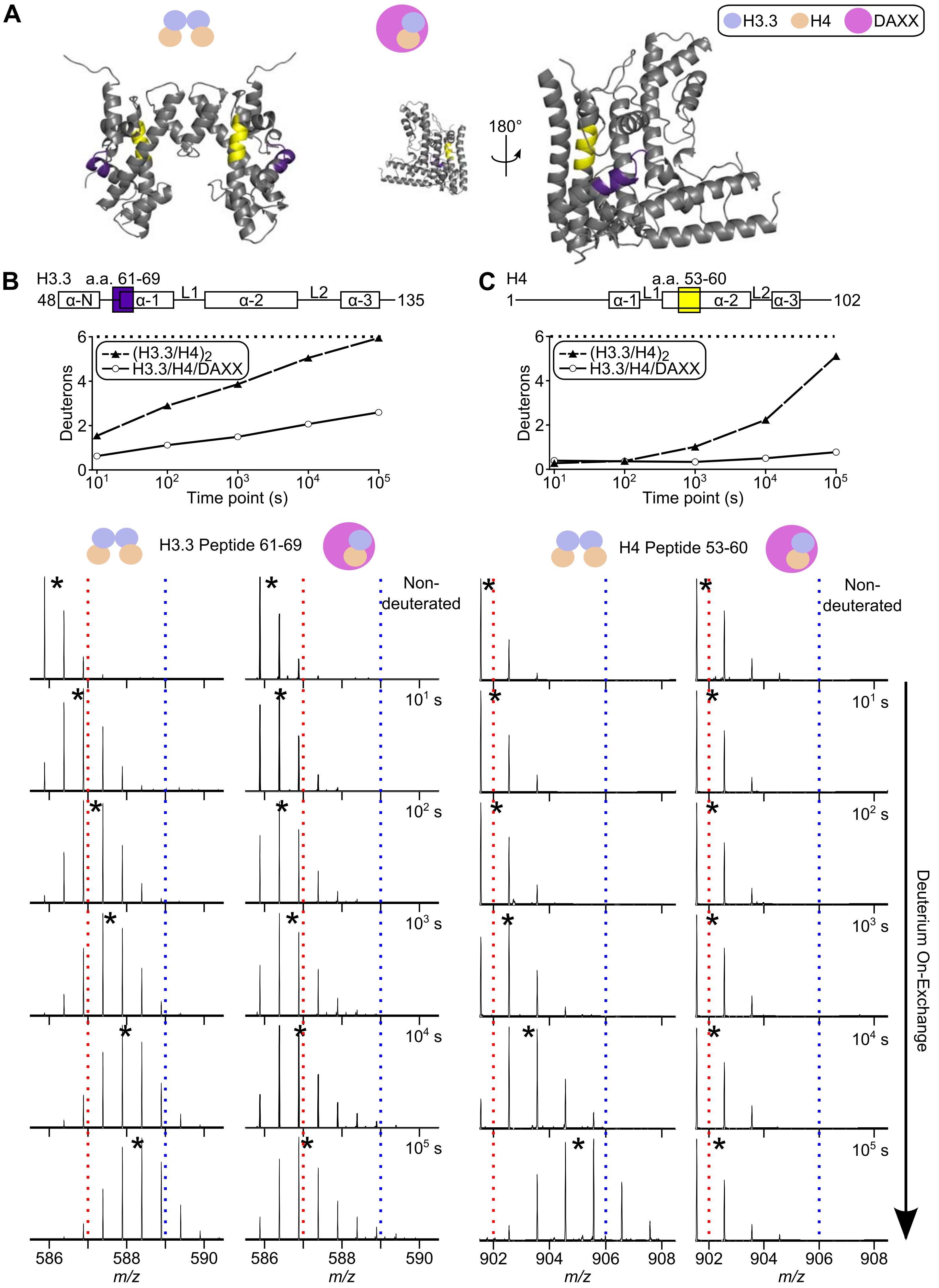
A
H3.3
H4
DAXX
180°
B
H3.3 a.a. 61-69
48 α-N α-1 L1 α-2 L2 α-3 135
(H3.3/H4)2
H3.3/H4/DAXX
Deuterons
Time point (s)
C
H4 a.a. 53-60
1 α-1 L1 α-2 L2 α-3 102
(H3.3/H4)2
H3.3/H4/DAXX
Deuterons
Time point (s)
H3.3 Peptide 61-69
H4 Peptide 53-60
Non-deuterated
10^1 s
10^2 s
10^3 s
10^4 s
10^5 s
Deuterium On-Exchange
m/z
586 588 590
902 904 906 908

DeNizio *et al.*, Supplemental Figure S2

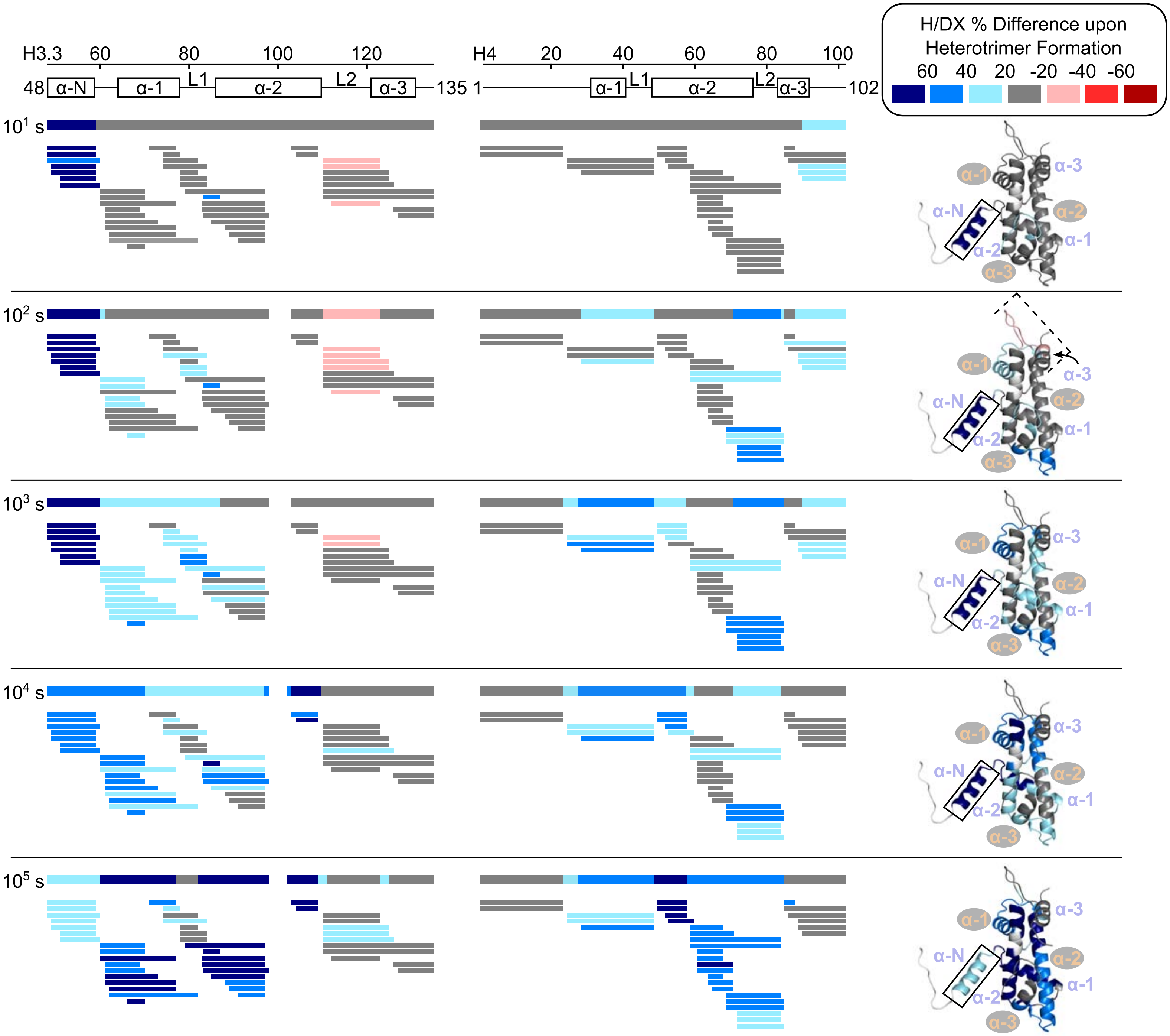

DeNizio *et al.*, Supplemental Figure S3

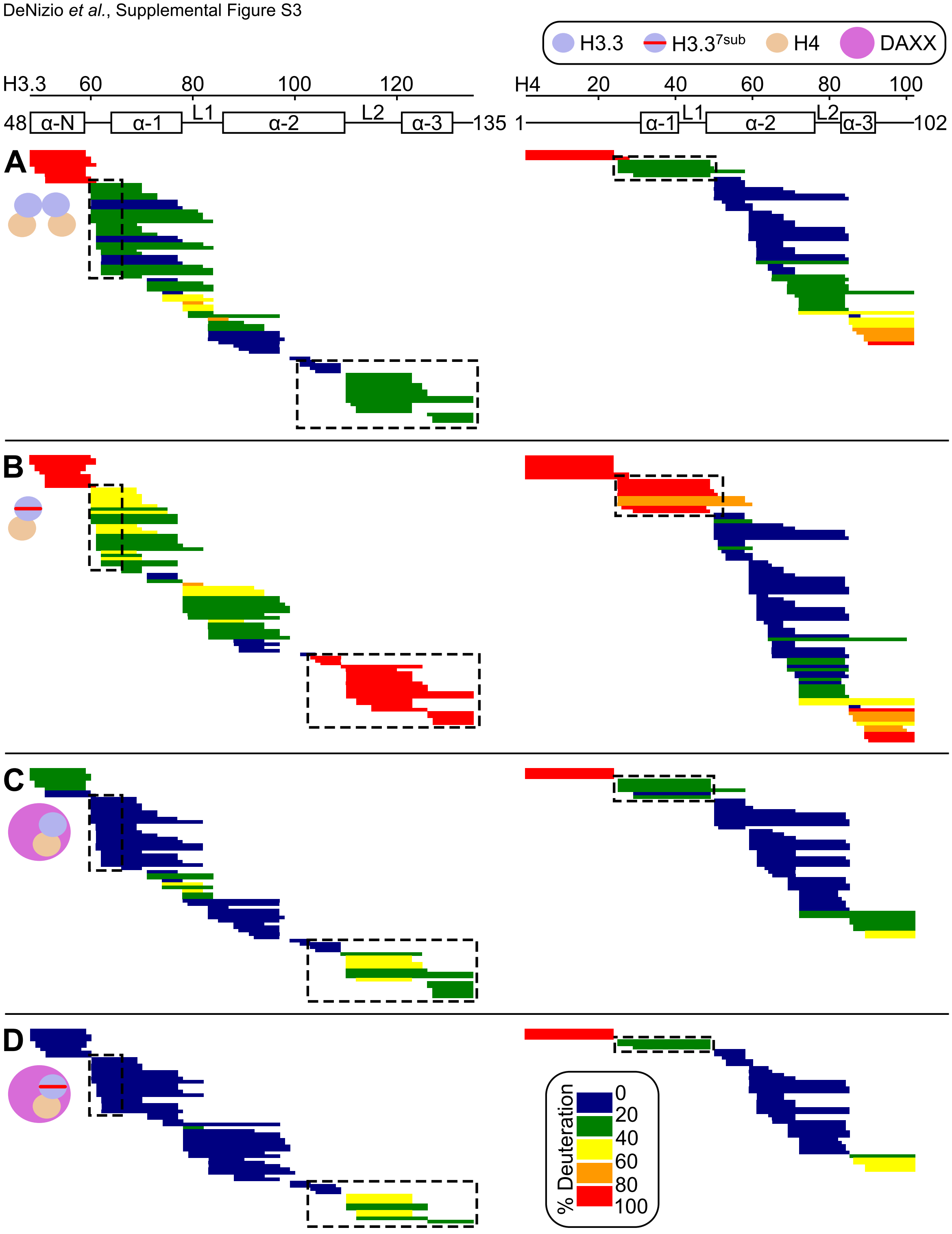

DeNizio *et al.,* Supplementary Figure S4

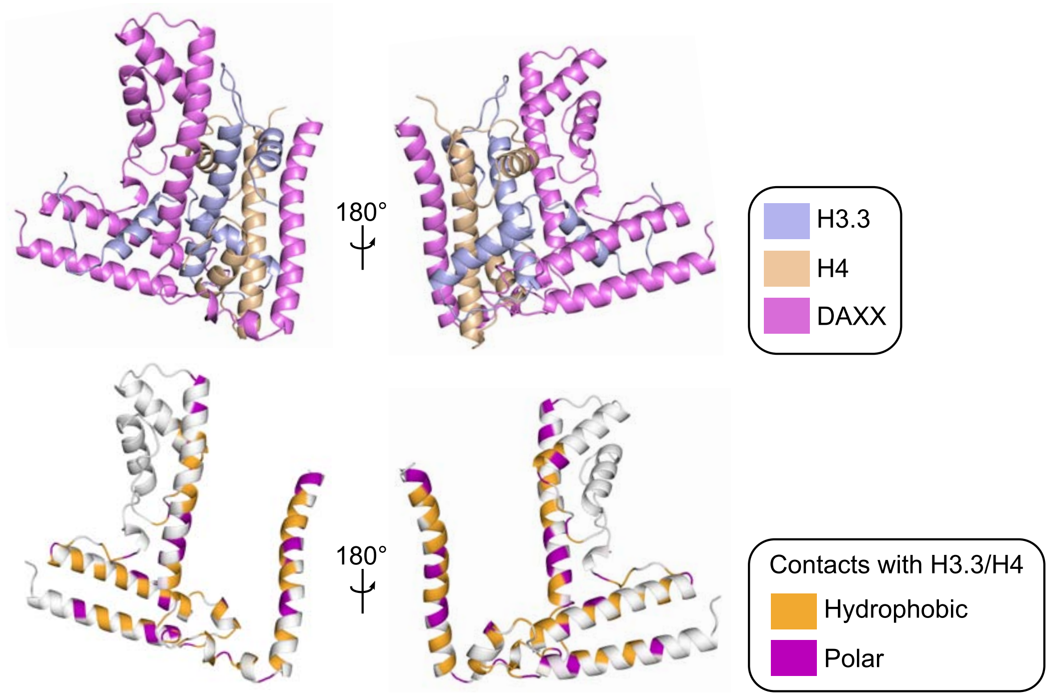

DeNizio *et al.*, Supplemental Figure S5

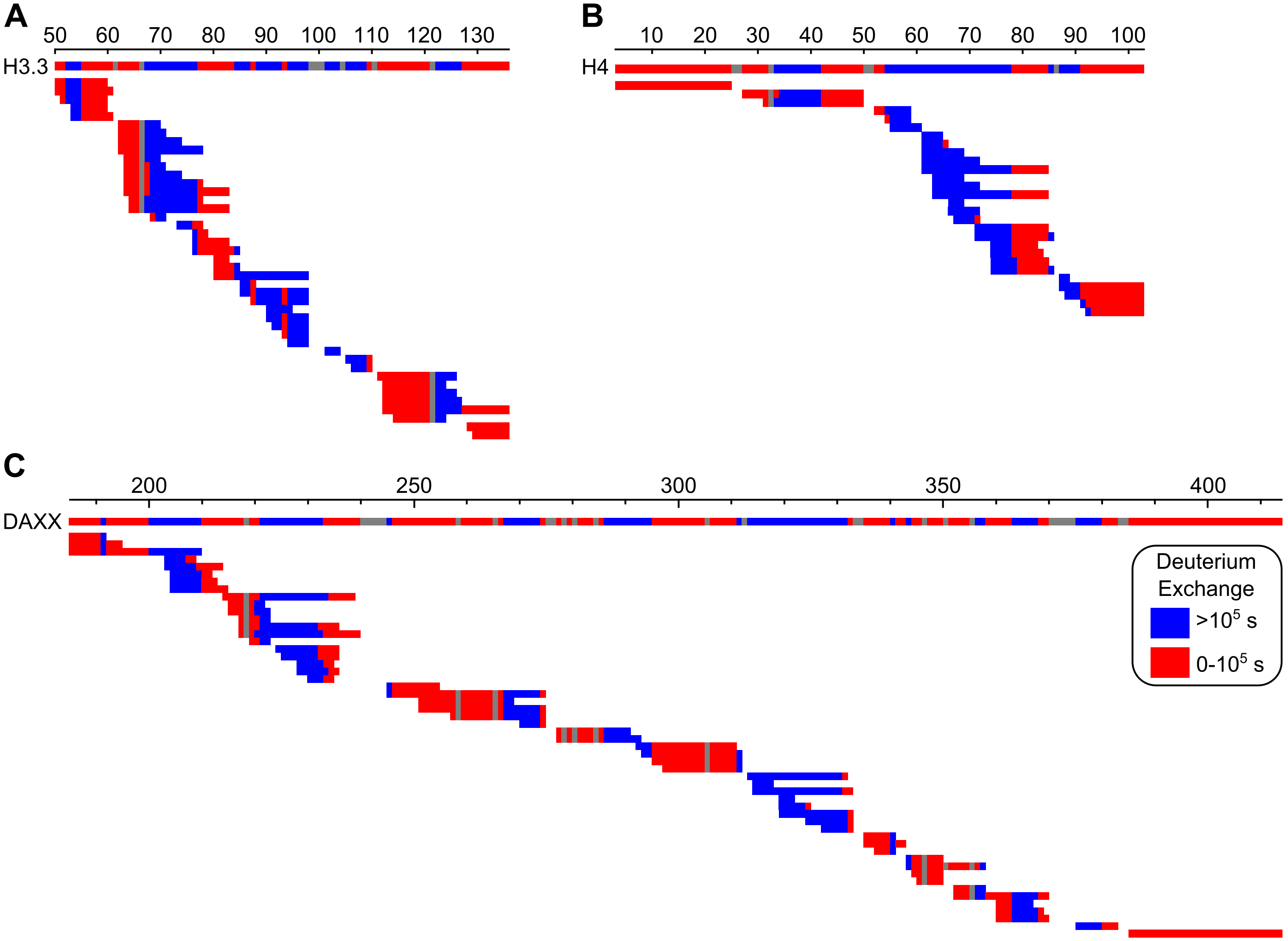

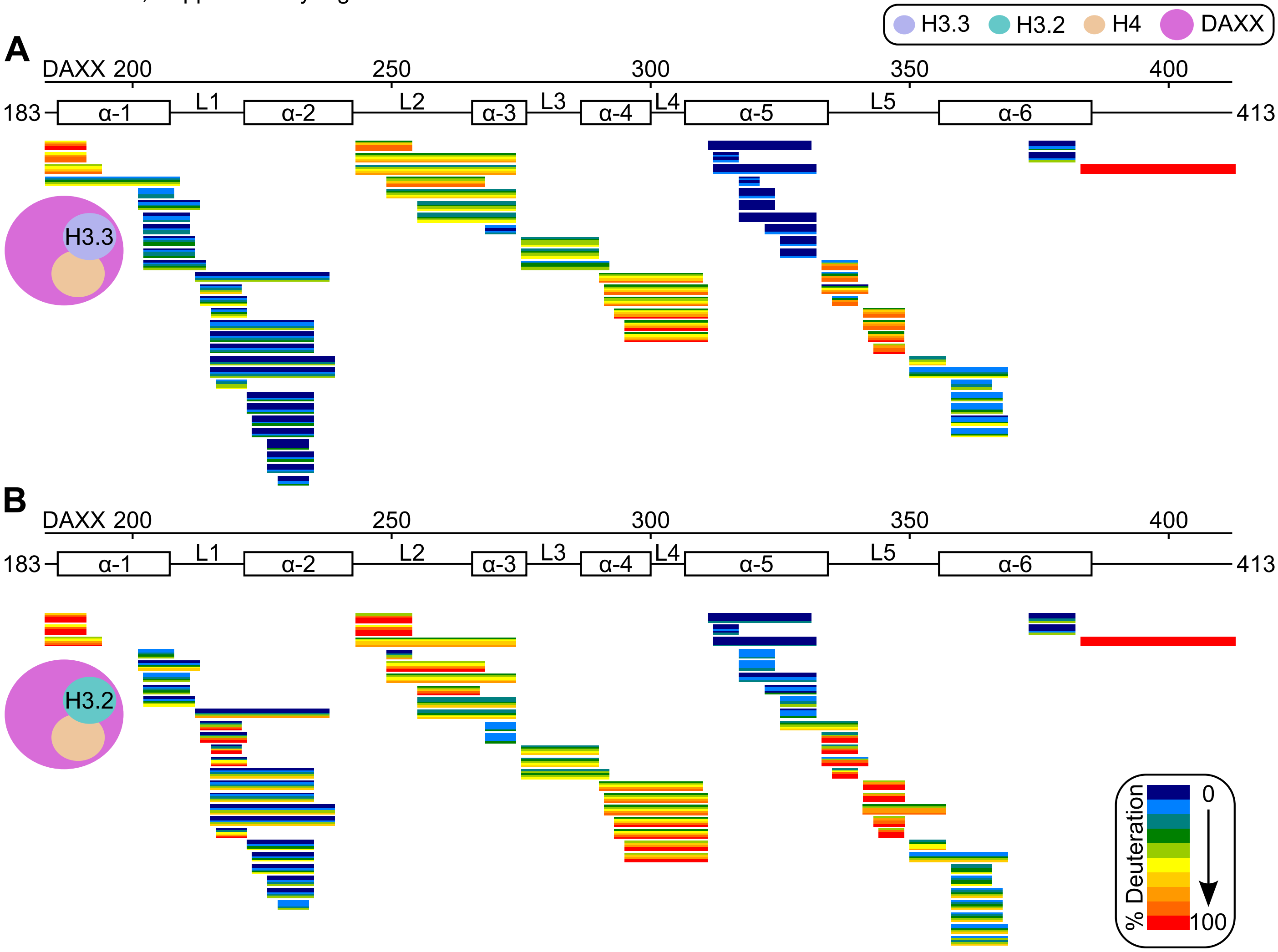
H3.3
H3.2
H4
DAXX
A
DAXX 200
250
300
350
400
183
413
α-1
L1
α-2
L2
α-3
L3
α-4
L4
α-5
L5
α-6
H3.3
B
H3.2
% Deuteration
0
100

DeNizio *et al.*, Supplemental Figure S7

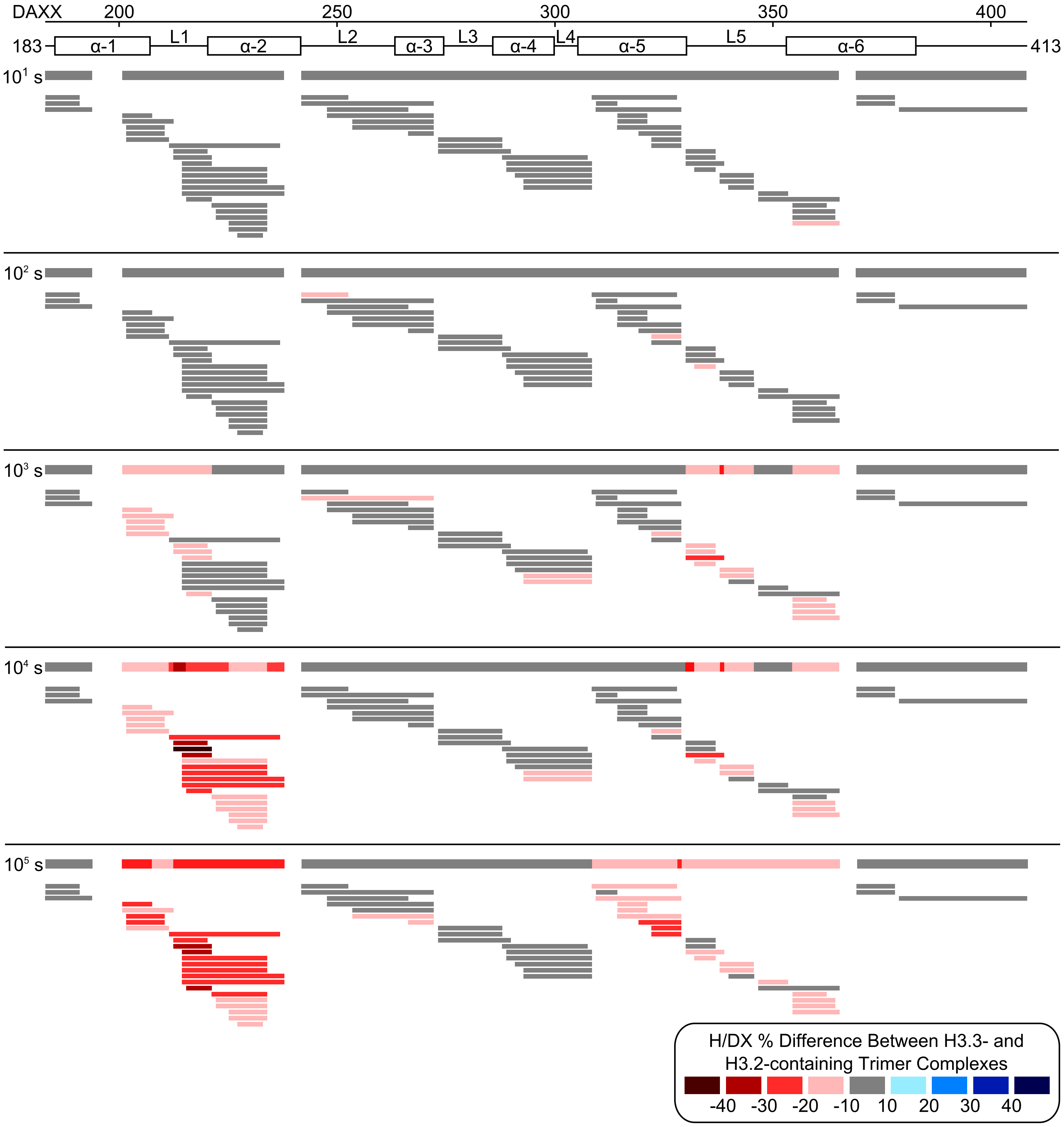

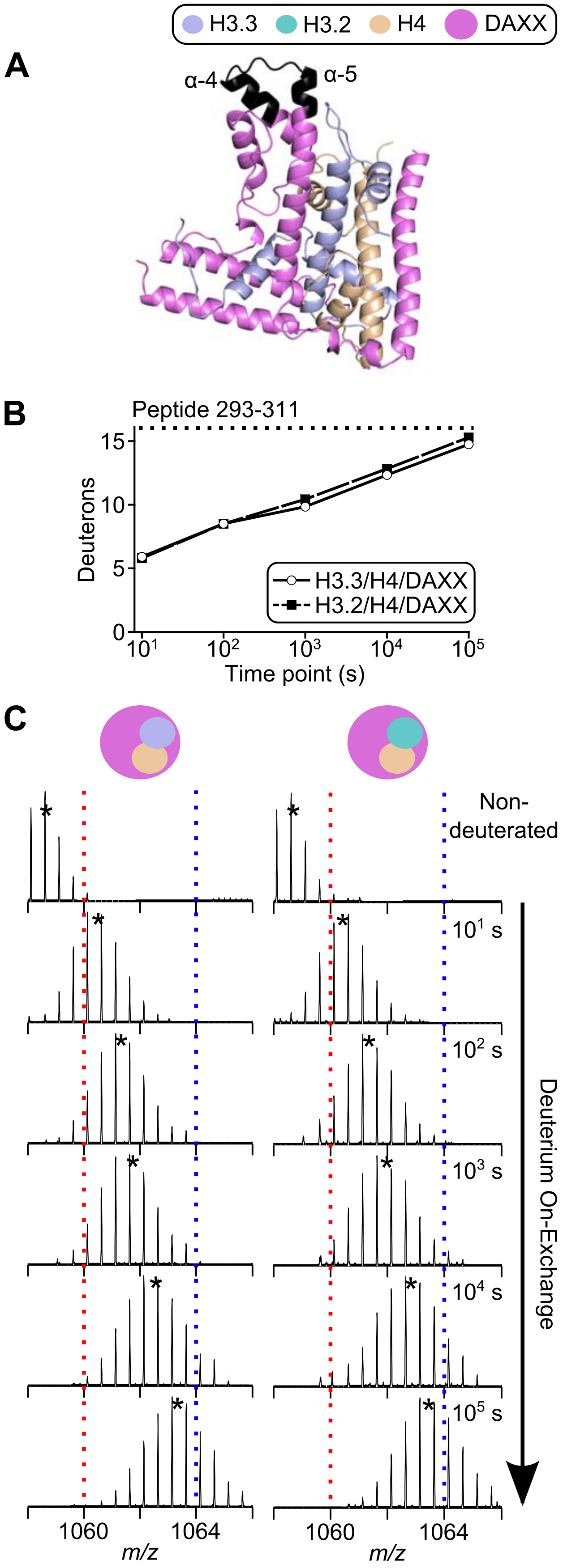
H3.3
H3.2
H4
DAXX
A
α-4
α-5
B
Peptide 293-311
Deuterons
15
10
5
0
H3.3/H4/DAXX
H3.2/H4/DAXX
$10^1$
$10^2$
$10^3$
$10^4$
$10^5$
Time point (s)
C
Non-deuterated
$10^1$ s
$10^2$ s
$10^3$ s
$10^4$ s
$10^5$ s
Deuterium On-Exchange
1060
1064
m/z

DeNizio *et al.*, Supplementary Figure S9

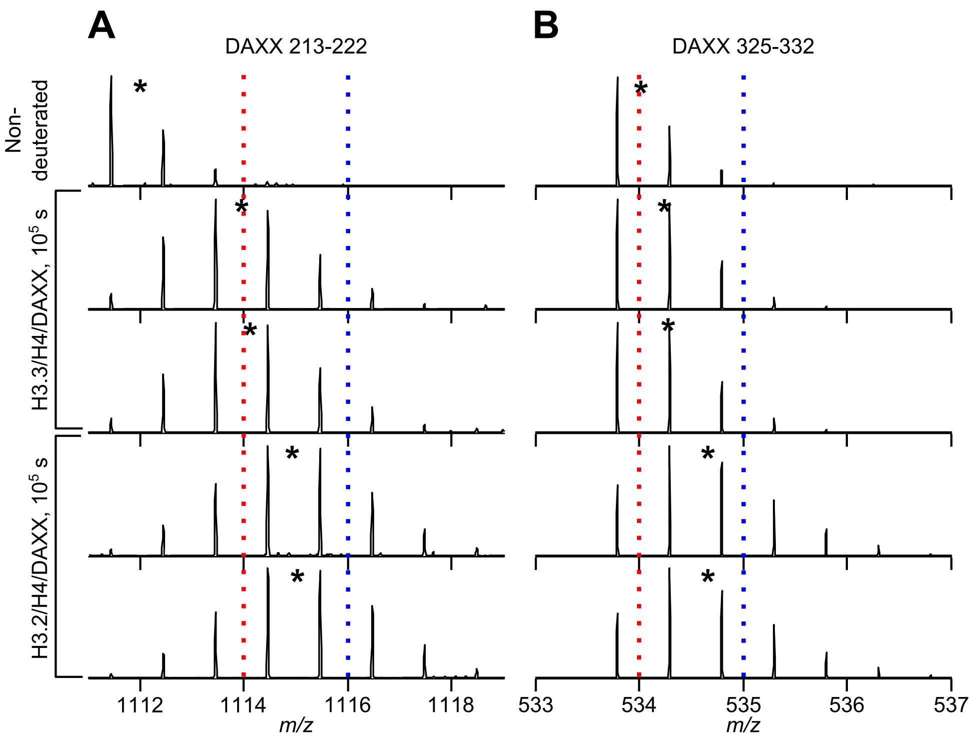

Supplement: Supplementary Data [file supp_gku090_nar-02623-m-2013-File009.pdf]
